# Supplementary material for: WordCommentsAnalyzer: A windows software tool for qualitative research
Source: F1000Res. 2018 Sep 4;7:536. Originally published 2018 May 3. [Version 2] doi: 10.12688/f1000research.14819.2 (PMC6137411; doi:10.12688/f1000research.14819.2)
Supplement: Supplementary file 3 [file f1000research-7-17614-s0002.tgz › b2a79496-4ada-47c4-9682-a99ce867836d.zip!/Eight Iranian Health Journals/Archives of Rehabilitation_61.docx]

© Archives of Rehabilitation. This work is licensed under CC BY-NC 4.0 (https://creativecommons.org/licenses/by-nc/4.0/).

Volume & Issue: دوره 5 - شماره 3

Title: بررسی تکرارپذیری مقادیر زمان تأخیری فعالیت واکنشی عضلات مچ پا در مقابل اغتشاش بیرونی ناگهانی در صفحه فرونتال

http://rehabilitationj.uswr.ac.ir/article-1-112-fa.html

هدف: هدف از این تحقیق تعیین تکرارپذیری زمان‌بندی فعالیت عضلانی مچ پا روی سیستم اغتشاش مچ پا در صفحه فرونتال تحت شرایط مختلف اغتشاش بیرونی است. زمان تأخیری شروع فعالیت، دامنه حداکثر فعالیت و زمان تأخیری حداکثر فعالیت عضلات مچ پا 10 مرد و زن سالم و 10 مرد و زن مبتلا به بی‌ثباتی مچ پا در یک مطالعه متودولوژیک مورد آزمون قرار گرفت. روش بررسی: زمان تأخیری شروع فعالیت، دامنه حداکثر فعالیت و زمان تأخیری حداکثر فعالیت عضلات مچ پا متعاقب اینورژن ناگهانی مچ پا توسط سیستم اغتشاش مچ پا و الکترومیووگرافی سطحی تعیین گردید. یافته‌ها: دامنه مقادیر ICC برای زمان تأخیری شروع فعالیت عضلات پرونئوس لانگوس (0/97-0/71)، تیبیالیس قدامی (0/99-0/75)، پرونئوس برویس (0/99-0/83) و سولئوس (0/99-0/97) در زوایای 10، 20 و 30 درجه در صفحه فرونتال است. این دامنه مقادیر برای دامنه حداکثر فعالیت عضلات پرونئوس لانگوس (0/99-0/81)، تیبیالیس قدامی (0/99-0/9)، پرونئوس برویس (0/99-0/64) و سولئوس (0/99-0/98) است و برای زمان تأخیری حداکثر فعالیت عضلات پرونئوس لانگوس (0/99-0/89)، تیبیالیس قدامی (0/99- 0/71)، پرونئوس برویس (0/99-0/91) و سولئوس (0/99- 0/69) به‌دست آمد. نتیجه‌گیری: تعیین زمان‌بندی فعالیت عضلات مچ پا به‌عنوان روش اندازه گیری تکرارپذیر ثابت گردید.

Title: مقایسه اثر محرکات صوتی بر مهار دگرسویی گسیل‌های صوتی گوش

http://rehabilitationj.uswr.ac.ir/article-1-113-fa.html

هدف: تحریک دگرسویی صوتی سیستم وابران، موجب مهار عملکرد سلول‌های مویی خارجی و کاهش دامنه گسیل‌های صوتی گوش می‌شود. در بررسی حاضر مقدار میانگین کاهش دامنه گسیل‌های صوتی برانگیخته با تن برست (TBEOAEs) با فرکانس مرکزی 2000 ،1000 و 4000 هرتز با ارائه دگرسویی صوت خالص و نویز باریک باند با فرکانس مرکزی 1، 2، 4 کیلوهرتز در گوش راست افراد 19 تا 27 ساله تعیین شده است. روش بررسی: این مطالعه بر روی 37 نفر (15 مرد و 22 زن) با شنوایی طبیعی انجام شد. پس از تطابق سایکوآکوستیکی، بلندی نویز باریک باند با صوت خالص در dBHL 70 در گوش چپ، پاسخ TBEOAEs با فرکانس مرکزی 1، 2 و 4 کیلوهرتز در گوش راست به‌دست آمد. سپس با ارائه دگرسویی صوت خالص70 دسی‌بل و نویز باریک باند با بلندی معادل، دامنه TBEOAEs اندازه‌گیری‌ شد. یافته‌ها: میانگین دامنه‌های TBEOAE با فرکانس مرکزی 2،1 و 4 کیلوهرتز با ارائه دگرسویی صوت خالص و نویز باریک باند کاهش نشان داد که کاهش نویز باریک باند در تمامی فرکانس‌ها بیشتر بود. همچنین میانگین دامنه‌ها در فرکانس‌های بالاتر کاهش کمتری نشان داد. نتیجه‌گیری: تحریک دگرسویی موجب کاهش دامنه TBEOAEs می‌شود و محرک دارای پهنای باند بیشتر، در ایجاد پدیده مهار دگرسویی کارآمد تراست. به‌نظر می رسد علاوه‌بر هماهنگی طیفی بین محرک تولیدکننده گسیل‌های صوتی گوش و محرک دگرسویی، پهنای باند فرکانسی نیز در کاهش دامنه گسیل‌های صوتی گوش و ایجاد پدیده مهار نقش بسزایی‌دارد.

Title: مقایسه اختلالات زبان در بیماران اسکیزوفرنیای پارانوئید و غیرپارانوئید

http://rehabilitationj.uswr.ac.ir/article-1-114-fa.html

هدف: این پژوهش با هدف بررسی اختلالات زبان در بیماران مبتلا به اسکیزوفرنیای مزمن پارانوئید و غیرپارانوئید در مرکز روان‌پزشکی رازی صورت گرفته است. روش بررسی: در این پژوهش با استفاده از آزمون زبان‌پریشی فارسی، اختلالات زبان در 40 بیمار مبتلا به اسکیزوفرنیا با نشانه‌های نوع مداوم (مزمن) که به دو گروه 20 تایی (پارانوئید و غیرپارانوئید) زن و مرد تقسیم شده بودند، بررسی و مقایسه شد. نمونه‌های مورد مطالعه در هر دو گروه از نظر متغیرهای جنس، سن، میزان تحصیلات، طول مدت بیماری، مدت اقامت در بخش‌های بستری‌دراز مدت، همتاسازی‌ شدند. مهارت‌های زبانی مورد ارزیابی‌ در آزمون فوق عبارت بودند از: روانی‌ گفتار آزاد، روانی‌ گفتار توصیفی، کیفیت گفتار آزاد، کیفیت گفتار توصیفی، درک شنیداری، بیان شفاهی، درک زبان نوشتاری، نوشتن، تکرار و میزان واژگان. داده‌های به‌دست‌آمده با استفاده از برنامه آماری SPSS برای ویندوز تحلیل آماری‌شد. یافته‌ها: یافته‌های حاصل از این پژوهش، نشان داد که در تمام بیماران، امتیاز کلی مربوط به مهارت‌های زبانی آزمون فوق، پایین‌تر از افراد سالم است و بیشترین کاهش مربوط به بخش‌های کیفیت گفتار توصیفی، روانی‌ گفتار توصیفی و میزان واژگان است. نتیجه‌گیری: بیماران پارانوئید در قیاس با بیماران غیرپارانوئیدی‌ در بخش‌های روانی‌ گفتار توصیفی، درک شنیداری، بیان شفاهی، میزان واژگان و امتیاز کلی آزمون، تفاوت معنی‌دار آماری نشان دادند. در بین متغیرهای‌ دموگرافیک، تنها متغیر میزان تحصیلات با برخی از بخش‌های آزمون ارتباط معنی‌داری را نشان داد.

Title: بررسی ارتباط شغل و دیسکوپاتی های‌ ستون فقرات

http://rehabilitationj.uswr.ac.ir/article-1-115-fa.html

هدف: اختلال در ساختمان و عملکرد دیسک (دیسکوپاتی) یکی‌ از شایع‌ترین علل کمردرد است. این بررسی با هدف تعیین نقش نوع شغل در ایجاد دیسکوپاتی‌ در بیماران بستری‌شده در بیمارستان امام سجاد(ع) نیروی‌ انتظامی‌ انجام شده است.  روش بررسی: این مطالعه روی پرونده پزشکی 240 نفر بیمار مبتلا به دیسکوپاتی‌ ستون فقرات که از سال‌های 1375 لغایت 1382 در بیمارستان امام سجاد(ع) ناجا بستری‌ شده بودند انجام گردید و اطلاعات در قالب یک مطالعه گذشته‌نگر توصیفی مورد بررسی قرار گرفت. یافته‌ها: دیسکوپاتی‌ستون فقرات گردنی، پشتی، کمری و بیشتر از یک ناحیه در رسته‌های خدماتی‌ انتظامی (%52/9) بهداری (%8/8) و اداری (%38/3) تفاوت چشم‌گیری داشت. بنابراین بررسی‌ ارتباط محل ضایعه با رسته خدمتی (0/028=P) معنی‌دار بود. ضایعه در سطح پشتی-کمری T12-L1) Thoracolumba) نیز با رسته خدمتی (0/013=P) رابطه معنی‌داری‌ داشت. بدین ترتیب که در رسته خدمتی‌ انتظامی %50 بیماران، در رسته خدمتی بهداری %37/5 و در رسته خدمتی‌ اداری %12/5 به دیسکوپاتی پشتی-کمری مبتلا بودند. نتیجه‌گیری: با توجه به شیوع بالای‌ دیسکوپاتی ‌در پرسنل نیروی‌ hنتظامی و معنی‌دارشدن ارتباط رسته خدمتی با محل ضایعه دیسکوپاتی و در جهت کاهش‌ هزینه‌های‌ درمانی و به‌حداقل‌رساندن طول مدت مرخصی، لازم است تمهیداتی ‌اندیشیده شود. ازآنجایی‌که تحقیقات مختلف تاثیر آموزش را بر کاهش مشکلات ستون فقرات ثابت کرده‌اند، می‌توان روش‌های‌ تصحیح وضعیت در موقعیت‌های مختلف شغلی، آموزش‌های ضمن خدمت و رعایت اصول ارگونومی (Ergonomic) را پیشنهاد نمود.

Title: همسرآزاری و عوامل مؤثر بر آن در زنان متأهل

http://rehabilitationj.uswr.ac.ir/article-1-116-fa.html

هدف: رواج همسر‎آزاری‌ در جوامع و تاثیرات سوء آن بر سلامت جسمی و روانی زنان و در نهایت تأثیر آن بر سلامت خانواده، در پژوهش‌های بسیاری نشان داده شده است. هدف پژوهش حاضر بررسی میزان شیوع همسر‎آزاری‌ روانی و جسمی و عوامل مؤثر بر آن در زنان متأهل شهر تهران بود. روش بررسی: نمونه آماری پژوهش حاضر را 1000 زن متأهل 18 تا 45 سال ساکن شهر تهران تشکیل می‌داد. نمونه آماری‌ دو گروه از زنان را شامل می‌شد: زنان گروه عادی (800=n) و زنان شاکی‌ که به‌دلیل مشکلاتی‌ که با شوهران خود داشتند به دادگاه خانواده مراجعه کرده بودند (200=n). ابزار پژوهش، پرسشنامه‎ای‌ است که از پرسشنامه‌ همسر‎آزاری موفیت و همکاران اقتباس شده بود. یافته‎ها: یافته‌های حاصل از پژوهش نشان داد که میزان همسرآزاری روانی و جسمی برای زنان عادی به‌ترتیب برابر با 87/9 و 47/9 درصد و برای زنان شاکی به‌ترتیب برابر با 99/5 و 91 درصد است. با افزایش سن زوجین و مدت زمان ازدواج، میزان همسرآزاری‌ افزایش می‌یابد. همسرآزاری با سطح تحصیلات زوجین (زن و شوهر) رابطه معنی‌دار معکوس دارد. هرچه تحصیلات زنان بیشتر باشد کمتر مورد آزار جسمی و روانی قرار می‌گیرند. همچنین هرچه میزان تحصیلات شوهران بیشتر باشد کمتر همسران خود را مورد آزار جسمی و روانی قرار می‌دهند. میزان همسرآزاری‌ در شوهرانی‌ که از الکل یا مواد مخدر استفاده می‌کنند بیشتر از شوهرانی‌ است که از الکل یا مواد مخدر استفاده نمی‌کنند. میزان همسرآزاری‌ در شوهرانی‌ که سابقه بیماری‌ روانی‌ دارند، بیشتر از شوهرانی‌ است که سابقه بیماری‌ روانی ندارند. میزان همسرآزاری جسمی و روانی‌ در زنانی‌ که به دادگاه‌های خانواده مراجعه کرده‎اند، بیشتر از زنان عادی‌ است. نتیجه‌گیری: همسرآزاری بسیار رایج است و با عوامل متعددی چون سن، میزان تحصیلات، اعتیاد شوهر به مواد مخدر، استفاده از الکل و سابقه بیماری‌ روانی وی‌ در رابطه است. تصویب قانونی جامع برای مقابله با همسر‎آزاری و افزایش آگاهی عمومی‌ درباره‌ همسر‎آزاری با استفاده از رسانه‎های جمعی می‌توانند در کاهش میزان همسرآزاری مؤثر واقع شوند.

Title: بررسی میزان موفقیت ارائه خدمت «آموزش در خانواده» طرح توان‌بخشی مبتنی‌بر جامعه به خانواده‌های دارای فرد مبتلا به معلولیت جسمی-حرکتی

http://rehabilitationj.uswr.ac.ir/article-1-117-fa.html

هدف: تعیین میزان اثربخشی خدمت «آموزش در خانواده» از مجموعه خدمات اصلی ارائه‌شده در برنامه توان‌بخشی مبتنی‌بر جامعه به افراد مبتلا به معلولیت جسمی-حرکتی و خانواده‌های آنها در شهرستان بابلسر بود. روش بررسی: برای این منظور 48 فرد مبتلا به معلولیت جسمی و حرکتی و خانواده‌های آنها از روستاهای ‌تحت پوشش طرح (گروه مورد) با 48 فرد معلول و خانواده‌های‌ آنها از روستاهایی‌که در همین شهرستان تحت پوشش طرح نبودند و از نظر سن، جنس، اندام درگیر، شدت نقص و میزان تحصیلات مراقب همتا بودند (گروه شاهد) در زمینه‌های آگاهی نسبت به عوارض بی‌تحرکی عضو، مهارت در‌ تغییر وضعیت‌دادن فرد، پیشگیری از جمود مفاصل و زخم بستر و همچنین کمک به فرد معلول در کسب استقلال در انجام فعالیت‌های‌ روزمره و حرکت در محیط و نیز نگرش آنان در مورد معلولیت و توان‌بخشی مورد بررسی قرار گرفت. اطلاعات موردنظر با مراجعه به درب منازل و پرسش از خانواده و فرد معلول و معاینه و مشاهده کسب و در فرم‌های مربوطه ثبت‌ و نمره داده شد و سپس مورد تجزیه و تحلیل قرار گرفت. یافته‌ها: تفاوت معناداری در میزان آگاهی افراد دچار معلولیت و خانواده‌های آنان بین دو گروه مورد و شاهد، در زمینه نحوه کمک به فرد معلول به‌منظور کسب استقلال در انجام فعالیت‌های‌ روزمره، جابه‌جایی و حرکت در محیط و عوارض بی‌حرکتی اندام‌ها و همچنین میزان مهارت آنان در زمینه کمک به فرد معلول برای‌ کسب توان انجام مراقبت‌های ‌شخصی و حرکت‌کردن در محیط به‌طور مستقل و نگرش ایشان در مورد معلول و معلولیت و اثربخشی‌ شیوه‌های ‌توان‌بخشی وجود نداشت (0/05>P)، اما مهارت افراد دچار معلولیت و خانواده‌های آنان در روستاهای ‌تحت پوشش طرح در زمینه نحوه پیشگیری از ایجاد خشکی مفاصل و زخم بستر و نیز چگونگی‌ تغییر وضعیت معلول به‌طور معنی‌داری بیش از گروه شاهد بود (0/04=P). نتیجه‌گیری: ارائه خدمت «آموزش در خانواده» طرح توان‌بخشی مبتنی‌بر جامعه در روستاهای شهرستان بابلسر تنها مهارت کاربرد تکنیک‌های توان‌بخشی غیرفعال در پیشگیری از عوارضی چون جمود مفاصل، زخم بستر و تغییر وضعیت دادن فرد معلول تأثیر مثبت داشته اما در مهارت خانواده و فرد معلول در استقلال‌یافتن معلول اثر معناداری نداشته است.

Title: بررسی میزان نیروی‌ گیرش جانبی‌ شست مددجویان کوادری‌پلژی‌ که از تحریک الکتریکی عضلات استفاده می‌نمایند با استفاده از شبیه‌سازی رایانه‌ای‌ یک مدل استاتیکی‌سه‌بعدی از دست

http://rehabilitationj.uswr.ac.ir/article-1-119-fa.html

هدف: مفصل انتهایی‌ شست دست مددجویان کوادری‌پلژی‌ که از تحریک الکتریکی عضلات جهت بازیابی حرکت انگشتان استفاده می‌نماید، هنگام عمل گیرش جانبی‌ دچار خمش بیش‌ازاندازه و در نتیجه گیرش ناموفق می‌گردد. در فعالیت‌های‌ روزمره این نوع گیرش جهت گرفتن و جابه‌جاکردن برخی از اجسام کاربرد فراوانی‌ دارد؛ لذا لازم است برای این دسته از مددجویان این امکان حرکتی بازیابی‌ گردد. در حال حاضر، مشکل فوق توسط تثبیت بند انتهایی‌ شست در امتداد بند میانی‌ که نیاز به عمل جراحی‌ دارد و منجر به ازدست‌دادن یک درجه آزادی می‌شود، رقع می‌گردد. در این تحقیق با استفاده از شبیه‌سازی‌ رایانه‌ای‌ یک مدل استاتیکی از دست، زاویه و نیروی‌ گیرش در روش متداول و روش‌های پیشنهادی جایگزین محاسبه و مقایسه گردید. روش بررسی: در مدل ارائه‌شده انگشتان، کف دست و جسم مورد گیرش به‌صورت صلب، ثابت و عمود بر صفحه افق و شست به‌شکل زنجیره‌ای از سه جسم صلب که توسط یک مفصل دو درجه آزادی به دست و دو مفصل یک درجه آزادی به یکدیگر متصل شده‌اند، در نظر گرفته شد. به‌ هر مفصل گشتاور مقاومتی، گشتاور فعال عضلات و گشتاور حاصل از نیروی خارجی بر بند انتهایی‌ شست اعمال گردید. عضلات فلکسور بلند شست (FPL: Flexor Pollicis Longus)، اکستانسور بلند شست (EPL: Extensor Pollicis Longus)، جمع‌کننده شست (ADP: Adductor Pollicis) و دورکننده شست (APB: Abductor Pollicis Brevis) در مدل به‌کاررفته شش روش متفاوت مورد بررسی قرار گرفت: 1- تحریک فلکسور بلند شست و فیوزکردن مفصل انتهایی‌ شست در زاویه صفر (روش متداول)؛ 2- در زاویه 15؛ 3- در زاویه 30 درجه؛ 4- تحریک فلکسور بلند شست؛ 5- تحریک هم‌زمان فلکسور بلند شست و EPL؛ 6- تحریک هم‌زمان فلکسور بلند شست، ADP و APB بدون فیوز نمودن مفصل. یافته‌ها: مقایسه مابین نتایج تجربی‌ دیگران و نتایج حاصل از مدل، مؤید اعتبار مدل است. یافته‌ها آشکار نمود استفاده از عضله فلکسور بلند شست بدون فیوزنمودن مفصل انتهایی‌ شست موجب گیرش جانبی نامناسب با نوک شست می‌گردد و فیوزنمودن مفصل در امتداد بند میانی، بدون ایجاد تغییر محسوسی‌ در نیروی‌ گیرش، زاویه گیرش را به شکل مؤثری بهبود می‌بخشد. اگرچه اضافه‌نمودن زاویه فیوز موجب افزایش ناچیز نیروی‌ گیرش می‌شود، زاویه گیرش به مقدار قابل توجهی‌ افرایش می‌یابد و حالت گیرش را نامناسب می‌نماید؛ لذا نتایج مدل مؤید اثربخشی‌ روش متداول است. نتایج نشان می‌دهد میزان گشتاور خمشی خلفی‌ آکستانسور بلند شست نمی‌تواند بر گشتاور خمشی قدامی فلکسور بلند شست غلبه نماید و تغییر قابل توجهی‌ در حالت گیرش ایجاد نماید، لذا به‌نظر می‌رسد این راه حل پیشنهادی موفقیت‌آمیز نباشد، اما در روش آخر، بدون نیاز به فیوزکردن مفصل، حالت گیرش تقریباً مشابه روش متداول و نیروی‌ گیرش 60 درصد بیشتر از نیروی‌ گیرش در روش متداول است. نتیجه‌گیری: در استفاده از تحریک الکتریکی عضلات می‌توان عمل گیرش جانبی‌ شست را بدون عمل جراحی و با استفاده از تحریک الکتریکی هم‌زمان عضلات فلکسور بلند شست، جمع‌کننده شست و دورکننده شست بازیابی نمود.

Title: بررسی و مقایسه مهارت‌های آگاهی واجی و سرعت نامیدن در کودکان نارساخوان و عادی

http://rehabilitationj.uswr.ac.ir/article-1-120-fa.html

هدف: در طی‌ دهه‌های‌ اخیر ارتباط میان نقص در مهارت‌های آگاهی واجی با مشکلات خواندن به‌طور وسیعی پذیرفته شده است و عمده‌ترین علت نارساخوانی، مشکلات پردازش واجی‌ در نظر گرفته شده است. اگرچه نتایج تحقیقات اخیر در زمینه سرعت نامیدن، حاکی از این است که این مهارت منبع احتمالی‌ دیگری جهت نارساخوانی است. پژوهش حاضر با هدف بررسی و مقایسه مهارتهای آگاهی واجی و سرعت نامیدن بر روی 58 کودک پایه دوم ابتدایی‌شهر تهران صورت گرفته است. روش بررسی: 28 نفر از آزمودنی‌ها نارساخوان و 30 نفر عادی بودند. تکالیف مورد استفاده در این تحقیق  آزمون آگاهی واج‌شناختی‌ دستجردی (1381) بود. کلیه اطلاعات جمع‌آوری‌شده در این پژوهش با استفاده از آزمون‌های‌ آماری‌ تی مستقل و یومن‌ویتنی مورد تحلیل قرار گرفتند، همچنین همبستگی میان مهارت‌های آگاهی واجی و سرعت نامیدن با استفاده از آزمون‌های ضریب همبستگی پیرسون و اسپیرمن در دو گروه آزمودنی‌ها مورد بررسی قرار گرفت. یافته‌ها: نتایج حاصل از اجرای آزمون‌های‌ آماری حاکی از آن است که بین دو گروه نارساخوان و عادی‌ در مهارت‌های مذکور تفاوت معنی‌داری وجود دارد و گروه نارساخوان در هر دو مهارت ضعیف تر از گروه عادی است. همچنین در این پژوهش میان مهارت‌های آگاهی واجی و سرعت نامیدن همبستگی معنی‌داری وجود نداشت. نتیجه‌گیری: نتایج این تحقیق نیز همسو با بسیاری از تحقیقات مشابه، نشانگر وجود مشکلات آگاهی واجی و سرعت نامیدن در افراد نارساخوان است. از نظر دیگر عدم همبستگی معنادار میان دو مهارت می‌تواند نشانگر آن باشد که سرعت نامیدن مستقل از آگاهی واجی‌ است.

Title: توان‌بخشی‌ گفتاری پس از ترمیم شکاف لب و کام

http://rehabilitationj.uswr.ac.ir/article-1-118-fa.html

علی‌رغم پیدایش روش‌های نوین ترمیم بیماران با شکاف لب و کام یا شکاف کام یا لب، بروز اختلال گفتاری‌ بیمار پس از ترمیم، دور از انتظار نیست. از هر دوهزار تولد، یک نوزاد دچار شکاف کام به‌تنهایی یا در هر هزار تولد یک نوزاد دارای‌ شکاف توأم لب و کام است. میزان شیوع این اختلالات نزد سفیدپوستان قدری‌ بالاتر از سیاه‌پوستان است و در منطقه آسیا با بروز بیشتری، حتی‌ به یک در هر پانصد تولد می‌رسد. ازآنجاکه امکان وجود سایر اختلالات در ناهنجاری‌ها در میان این نوزادان وجود دارد؛ لذا بررسی‌ کامل تاریخچه‌ای و اخذ معاینات کامل بالینی‌ آنها پس از تولد ضروری‌ است. بررسی مسائل ارثی‌‌بودن این ناهنجاری‌ها با توجه به تاریخچه ازدواج‌های خانوادگی یا احتمال بروز ناهنجاری‌های مشابه در سایر فرزندان نیز ضروری‌ است. بروز شکاف کام به‌تنهایی، بیشتر از شکاف توأم لب و کام است که به‌همراه ناهنجاری‌های‌ دیگر به‌صورت نشانگان (Syndrome) به‌وجود می‌آیند. بنابراین، بررسی‌ این‌گونه بیماران با نشانگانی‌ که تقریباً یک سوم کل بیماران با شکاف کام تنها را تشکیل می‌دهند، می‌بایست از دیدگاه‌های مختلف بالینی‌ مـورد تأکیــد قرار گیرد. شایع‌‌‌ترین نشانگان همـراه با شــکاف کــام تنها «نشانگان پیر رابین» (Pierr Robin) است که علاوه‌بر شکاف کام، علائم دیگری چون کوچکی فک تحتانی و بیرون زدگی زبان را به‌همراه دارند. نحوه شکاف کام تنها در بیماران نشانگانی‌ به شکل V و در بیماران به‌همراه نشانگان به صورت U است. پس از بررسی‌ کامل عمومی نوزاد، وضع تغذیه‌ای وی از جهت جلوگیری از سوء تغذیه، کاهش وزن و عدم رشد جسمی ضروری‌ است. در صورت فراهم‌بودن کلیه شرایط سلامتی، عمل جراحی‌ ترمیمی لب و هر دو بخش سخت و نرم کام، در اولین فرصت ممکن صورت می‌گیرد. در شرایطی‌ که شکاف لب ناوسیع باشد، می‌توان ترمیم کام را در سن زیر 6 ماهگی و در صورت ‌همراه‌بودن با شکاف کام، زیر 18 ماهگی، مورد بررسی قرار داد. ازآنجاکه در عمل جراحی مزبور میزان قابل توجهی از اعصاب، مخاط، عضلات و قطعات استخوانی مورد دستکاری قرار می‌گیرد؛ لذا قبل و پس از ترمیم نواقص، کارایی‌ کامل و مناسبی‌ را در این اجزا نمی‌توان انتظار داشت. در نواحی‌ آناتومیکی‌ یادشده، مجموعه اجزای لب و کام که در نواحی خلف دهان، با انقباض عضلات و بالارفتن، موجب انسداد راه بینی‌ گردیده یا با شل‌شدن خود موجب بازشدن راه حلقی و بینی می‌شوند، دچار اختلال می‌گردد. میزان بروز این‌گونه اختلال در %20 از کل بیماران تحت عمل جراحی‌ ترمیمی لب و کام دیده می‌شود. اختلال مزبور، سبب اختلال عملکرد دریچه کامی-حلقی‌ می‌شود و خود سبب اختلال «پر خیشومی‌شدگی» (Hypernasality) یا «کم خیشومی‌شدگی» (Hyponasality) گفتار می‌گردد. بر همین اساس در جراحی‌های‌ ترمیمی لب و کام، مشاوره و مداخله آسیب ‌شناس گفتار و زبان، از اصولی ‌بسیار با ارزش و مهم تلقی می‌گردد. گرچه تبادل نظر و کار گروهی ‌با سایر متخصصین نیز در صورت لزوم، از اهمیت ویژه‌ای‌ در گروه‌درمانی‌ این بیماران نیز محسوب می‌گردد.

Title: زخم فشار و راه‌های پیشگیری از آن

http://rehabilitationj.uswr.ac.ir/article-1-1827-fa.html

زخم فشار یا زخم بستر یک آسیب یا ضایعه در پوست و بافت‌های زیرین آن محسوب می‌شود و معمولاً به‌دنبال فشار غیرقابل رفع بر نسوج ایجاد می‌شود و در افراد پیر و کسانی که در مراحل بحرانی بیماری هستند یک مشکل عمده بوده و باعث ایجاد درد، کاهش کیفیت زندگی و افامت‌های طولانی در بیمارستان می‌گردد. پیشگیری و درمان زخم برای بهبود و ارتقای وضعیت تندرستی بیمار از راهکارهای مقرون‌به‌صرفه به‌شمار می‌آید. هرگاه در مدت زمانی بیش از 2 ساعت، فشار موجود بر عروق کاپیلری شریان‌ها در نقطه‌ای به 70 mm-Hg (بیش از 2 برابر فشار کاپیلری شریان‌ها) برسد زخم فشار یا زخم بستر ایجاد خواهد شد که برحسب عمق گرفتاری به 4 نوع تقسیم می‌شود. مهم‌ترین نکته در پیشگیری از ایجاد زخم بستر یا زخم فشار، کاهش فشار از آن نقطه موردنظر است که بهترین کار برای کاهش و رفع فشار جابه‌جاکردن بیمار است. در صورت بروز زخم فشار می‌بایست برحسب نوع زخم، درمان مربوطه (پانسمان، آنتی‌بیوتیک‌تراپی، دبریدمان و جراحی) را انجام داد.
